# Perceptions of fairness, inclusion and safety: the differential impact of contrasting experiences on academics and professional services staff

**DOI:** 10.1007/s10997-024-09721-z

**Published:** 2024-10-12

**Authors:** Mariana Pinho, Belinda Colston

**Affiliations:** 1 Centre for Environmental & Marine Studies, Department of Biology, University of Aveiro, Campus Universitário De Santiago, 3810-193 Aveiro, Portugal; 2 Eleanor Glanville Institute, University of Lincoln, Lincoln, UK

**Keywords:** Academic staff, Professional services staff, Organizational inclusion, Psychological safety, Organizational commitment

## Abstract

The current study explores how organizational dimensions relate to and impact organizational commitment, comparing staff in academic positions with staff in professional services roles. Data was collected from 281 academic and 294 professional services staff within university environments who completed extensive questionnaires. Overall academics manifested lower levels of emotional attachment to, and perceived obligation to remain in their university, felt less safe to take interpersonal risks, to speak up and lower support for their work-life balance than their professional services colleagues. The perception of procedural fairness and discrimination impacted academics loyalty and felt obligation to remain and reciprocate organizational investments to a higher extent. Psychological safety positively influenced staff’s commitment. Emotional and obligation-based types of commitment were more strongly affected by psychological safety among academics than professional services staff. Finally, work-life balance support positively influenced staff’s commitment, appearing to be an equally important dimension to drive emotional and obligation-based types of commitment from both academics and professional services staff. This study brings important contributions to research on the working environment of academics and professional services staff and indicates that development of clear policies to promote and maintain fairness, psychological safety and work-life balance, together with active monitoring and evaluation of their impact, should be a key focus for higher education institutions.

## Introduction

Higher education institutions face the growing challenge of employing and retaining talent. Identifying the organizational components that influence engagement and dedication will aid the sector to foster organizational commitment among all staff and improve performance. Given the recency of interest in viewing universities as organizational environments, there is little research on the working experiences of professional services staff, particularly in comparison to their academic colleagues. Despite the number of professional services staff working in the UK higher education sector and their level of responsibility have drastically increased over the last decade (Wolf & Jenkins, 2021), a clear shortage of research analyzing their experiences remains (Tight, 2021). Most research to date has focused on role variation or professional identity of professional services staff rather than their work environment and commitment to the organization. Therefore, the inclusion of professional services staff and comparison with academic staff remains important, illuminating the unique experiences of university staff in these different roles, and the wider impact on the organization.

In the present study, professional services staff refers to employees responsible for the provision of professional and administrative support services to university staff, students and sometimes to the wider public. Academic staff includes personnel whose primary assignment is research.

The higher education sector has become increasingly target-driven and market-led, with the adoption of quality assurance systems to measure academic staff’s performance (e.g. Richards et al., 2016a, 2016b; Sang et al., 2015). The Research Excellence Framework (REF) and Teaching Excellence and Student Outcomes Framework (TEF) are examples of such systems in the UK. Consequently, organizational culture and behavior have shifted and negatively impacted academics’ health and well-being (e.g. Camilleri & Camilleri, 2018; Smith & Ulus, 2020). A growing body of research has demonstrated the profound effect of those changes (e.g. Smith & Ulus, 2020) in a population with already higher propensity to develop mental health issues (Guthrie et al., 2018; Kinman & Wray, 2013). Namely, current higher education organizational culture, working conditions and the increasing number of roles staff is expected to preform are linked to high levels of stress, chronic burnout, anxiety, job dissatisfaction and work-life conflict among academic employees (Barkhuizen et al., 2014; Guthrie et al., 2018; Hogan et al., 2016; Richards et al., 2016a, 2016b; Simons et al., 2019; Zábrodská et al., 2018) (for a review on academics’ mental health see Urbina-Garcia, 2020). An international comparison of national higher education systems indicated that academics in the UK have one of the lowest levels of job satisfaction and feel stressed with the performance-based management (Shin & Jung, 2014). With contemporary academic careers becoming highly competitive, with more precarious working conditions, academics are often forced to move institution for promotion or attaining a secure post (Richardson et al., 2019; Sierkierski et al., 2018).

Professional services staff, on the other hand, tend to have more ‘traditional’ and linear career paths (Gander et al., 2019; Veld et al., 2016). Due to the distinct nature of the roles, academic staff tend to develop their identity and loyalty around their subject area and the pursuit of individual goals (e.g. publications, funding), while professional services staff have their identities associated with their position and generally their focus is more on the delivery of organizational and operational objectives (Collinson, 2006; Coomber, 2019; Kuo, 2009; Whitchurch, 2010). Previous studies have shown that academics are significantly more likely than professional services staff to report higher levels of stress at work, less favorable perception of working conditions, lower perceived participation on decision making at work, and lower levels of commitment to the organization (Fontinha et al., 2019; Tytherleigh et al., 2005). Nonetheless, research has found professional services staff feel “invisible”, due to lack of value associated with their work (Caldwell, 2022; Simpson & Fitzgerald, 2014; Szekeres, 2011). They also reported lack of time, financial and line-manager support for undertaking professional development for career progression and inequity of treatment compared to academics (Coomber, 2019; Holmes, 2020).

Most of the studies to date have focused on either analyzing the experiences of academic staff (Nikunen & Lempiäinen, 2020; Richards et al., 2016a, 2016b) or professional services staff separately (e.g. Gander, 2018; Gander et al., 2019; Rush & Olivier, 2021), or have made limited comparisons between the two in terms of well-being and working conditions (e.g. Fontinha et al., 2019; Johnson et al., 2019). Therefore, much less is known about the operation and differential impact of organizational practices and priorities on both academic and professional services staff, and yet policies and practices continue to be shaped without due consideration of both roles and their unique experiences. A recent study found that 60% of university staff in the UK manifested their intention to leave the sector in the next five years (UCU, 2022) and workplace culture has been identified as one of the primary reasons why staff leaves academia (Spoon et al., 2023). With turnover increasing for academics and professional services staff (CUPA-HR, 2023), it is vital to understand the mechanisms underlying and influencing organizational culture in order to improve the quality of staff’s experience, their well-being and consequently contribute to retention in higher education sector.

The current study explores how organizational fairness and inclusion, psychological safety and support for work-life balance relate to organizational commitment, comparing staff in academic positions with staff in professional services roles. It aims to strengthen our understanding of the processes through which different organization related dimensions influence commitment and to reveal how individuals’ experience of organizational inclusion, fairness and safety can be improved, leading to more equitable work environments and advance systemic change in higher education.

### Theoretical development

Conservation of resources theory (Hobfoll, 1989, 2001) explains that individuals strive to obtain, build, protect, and foster resources. Resources can be objects, personal characteristics, conditions, energy and the methods to acquire them. According to the theory, stress occurs when there is a threat to loss of resources, when resources are lost or when resources are not acquired after significant effort has been made (Hobfoll et al., 2018). It presents a framework to comprehend the effect of the organizational environment on staff’s behavior (Hobfoll et al., 2018). Based on the conservation of resources model our study explores how organizational culture and perceived work conditions impact organizational commitment of staff working in higher education. Organizational commitment widely refers to an employee’s attachment, identification, and involvement with the organization (Mowday et al., 1979). Previous research has found that workplace culture, including supportive and fair policies are crucial to resource preservation and enhancement, leading to lower intentions to quit, higher job satisfaction and engagement (e.g. Au & Ahmed, 2016; Clark et al., 2017; Janssen et al., 2010; Rofcanin et al., 2017).

### Organizational commitment, perceptions of fairness and inclusion

Organizational commitment plays an important role in organizational behavior and culture. For example, organizations with highly committed employees achieve superior long-term performance and have lower turnover (e.g. Kim, 2005; Meyer et al., 1993; Tetteh et al., 2020). Therefore, enhancing organizational commitment among staff is an essential element as it will translate into higher retention, improved performance, and continuity in the organization’s area of expertise.

Organizational inclusion refers to the perception that one’s unique contribution to the organization is valued and participation embolden (Mor Barak, 2015; Nishii, 2013; Shore et al., 2011). Organizational fairness, on the other hand, is defined as one’s perception about the process and outcome being impartial, meaning that decisions, processes and practices are based on individual merit and performance rather than one’s social identity (e.g. gender, race, etc.) (Derks et al., 2007; Mor Barak et al., 1998). Previous research has found that organizational inclusion and fairness positively affect outcomes at the individual, group and organizational level (e.g. innovation, higher job satisfaction, increased employee retention, improved performance and wellbeing) and are associated with higher levels of organizational commitment (Colquitt et al., 2001; Findler et al., 2007; Mor Barak et al., 2016).

Organizational fairness and inclusion can be considered major elements of social support that according to the conservation of resources theory critically influences how resources are attained and influences organizational commitment (Hobfoll et al., 2018). Using Meyer and Allen’s model of commitment (Meyer & Allen, 1990), two areas of commitment will be explored in the current study: affective commitment that denotes an emotional attachment to, identification with and involvement with the organization; and normative commitment which pertains to a perceived obligation to remain in the organization (Meyer et al., 1993). Therefore, it is hypothesized that organizational fairness will positively influence staff’s affective (H1a) and normative commitment (H1b). Similarly, organizational inclusion will positively influence staff’s affective (H2a) and normative commitment (H2b). Meaning if staff perceives the organizational environment to be fair and inclusive and as the organization caring and valuing them, this will have a positive influence on their emotional attachment and obligation to remain at the organization.

### Psychological safety and organizational commitment

Psychological safety is generally defined as a shared belief that the workplace is safe for interpersonal risk taking (Edmondson, 1999, 2018). It allows staff “to feel safe at work in order to grow, learn, contribute, and perform effectively in a rapidly changing world” and be able to express their true self without fear (Edmondson & Lei, 2014; Kahn, 1990).

If a work environment has high psychological safety, it is easy for staff to acknowledge their mistakes, articulate their ideas, and make contributions without fear of judgement, retribution, or humiliation (Edmondson, 1999, 2018). According to the conservation of resources theory, individuals with greater resources are less vulnerable to resource loss and more able to organize resource gain through using their existing resources (Hobfoll, 2011). Therefore, if staff feels safe, they can utilize all their available resources to meet job demands and achieve their professional goals.

Psychological safety has been shown to influence work performance, effectiveness (Edmondson & Lei, 2014; Schaubroeck et al., 2011), creativity and innovation (Madjar & Ortiz-Walters, 2009; Newman et al., 2017). A psychologically safe environment also brings benefits that influence work engagement and organizational commitment (Brimhall et al., 2017; Christian et al., 2011; Frazier et al., 2017; Singh & Winkel, 2012; Singh et al., 2013). Psychological safety is considered, according to the conversation of resources theory, a resource, that can prevent staff from resource loss or depletion (Kızrak et al., 2024). Consequently, it hypothesized that higher levels of psychological safety expressed by staff will positively influence affective commitment (H3a) and normative commitment (H3b).

### Work-life balance support, employee retention and commitment

Over the last decades, the struggle to find the right balance between fulfilling work and non-work responsibilities has been amplified in many countries (Sullivan, 2019). Work-life balance is defined as “the individual perception that work and nonwork activities are compatible “(Kalliath & Brough, 2008). It remains an important factor in employee satisfaction, retention and commitment to the organization (e.g. Kalliath & Kalliath, 2015; Weale et al., 2019). For example, a healthy work-life balance has been shown to mediate job satisfaction, commitment, and a feeling of safety at work (Weale et al., 2019).

Support for work-life balance is gained through the organizational culture, managers’ and colleagues’ support (Dikkers et al., 2004; Thompson & Prottas, 2006). Managers hold one of the most important positions regarding support for work-life balance, as they not only transmit the organization’s culture, but they also signal their own norms by giving or withholding support for employees’ non-work responsibilities (Hochschild, 1997; Thompson et al., 1999). Employees with more supportive managers experience lower levels of work-life conflict (van Breeschoten & Evertsson, 2019). Consequently, managers’ awareness of the importance and impact of their role is vital in promoting a truly supportive environment for all staff.

As academia has shifted its culture to a more target-driven and managerialist approach (Kinman & Wray, 2014; Richards et al., 2016a, 2016b; Sang et al., 2015), being an ‘ideal academic’ has shifted towards longer working hours, being willing and able to travel, being research active and productive, and being embedded within social networks that will enhance promotion prospects (Sang et al., 2015). These demands have consequences for those who cannot meet them (e.g. staff with caring responsibilities, disabilities) and have a big impact on work-life conflict. Recent research revealed that when compared to professional services staff, academics are at a greater risk of work overload and work-life conflict (Johnson et al., 2019). Furthermore, academics who perceive to have a healthy work-life balance have more favorable perceptions of their working conditions and have a higher level of commitment to their organization (Fontinha et al., 2018). The conservation of resources theory views work-life balance as a resource and positively relates it to the absence of stress. Drawing on the conservation of resources theory, it is hypothesized that staff’s perceptions of greater work-life balance support will positively influence affective commitment (H4a) and normative commitment (H4b).

### Higher education sector in the UK

Our hypotheses were tested in a sample of academic and professional services staff working at British universities where performance culture has had an enormous impact on education policy (Teichler, 1988). UK universities are simultaneous public institutions and private enterprises and represent 1.2% of the UK's GDP (UUK, 2017). In 2020−2021, the higher education sector in the United Kingdom employed 415,970 members of staff (224,530 academic staff and 191,440 professional services staff) and had approximately 2.75 million students enrolled (HESA, 2022a, 2022b). The UK higher education sector remains a global leader and most research produced by UK universities is considered world leading or internationally excellent (REF, 2022). The evaluation of research quality has invigorated the perception of managerial approaches as indispensable to universities’ performance and has changed universities’ culture (Yokoyama, 2006). To maintain their world-leading status, the UK higher education sector shifted its culture to a more target-driven and managerialist approach, becoming more focused on financial sustainability and customer centricity (e.g. Richards et al., 2016a, 2016b; Sang et al., 2015). Organizational culture and staff at British universities, therefore, have been impacted by those changes. Academic and professional services staff are constrained by complex internal and external factors that might differ across roles and distinctly affect their working conditions and commitment. It is, therefore, important to understand those factors and their impact on different roles to improve staff management, retainment and the design of targeted organizational policies.

## Methodology

### Participants

Data were collected from 575 members of staff (244 female, 116 male, and 215 not disclosed) at six UK universities as part of a larger research project on inclusive research environments. The sample was constituted by 281 (48.9%) academic staff, and 294 (51.1%) professional services staff.

Table 1 lists the participants’ socio-demographic characteristics. Participants’ employment duration at their current university ranged from one week to 39 years. The largest populated participant age group was 35−44 years of age (31.2%), 80% had a partner or spouse, and 45% indicated having caring responsibilities for dependent children or adults. Of those with caring responsibilities (*n* = 220), 55 had children under 6 years (25.0%), 103 had a child between 6 and 18 years old (46.8%), 16 were responsible for young adults (7.3%) and 46 cared for dependent adults (20.9%).

**Table 1:** Demographic characteristics of the participants

	Academic	Professional services	Total
	*n* (%)	*n* (%)	*n* (%)
	281 (49%)	294 (51%)	575 (100%)
Employment duration (years)			
< 1	24 (8.6%)	43 (14.7%)	67 (11.7%)
1 − 5	118 (42.4%)	147 (50.2%)	265 (46.4%)
6 − 10	66 (23.7%)	48 (16.4%)	114 (20%)
11 − 15	31 (11.2%)	20 (6.8%)	51 (8.9%)
16 − 20	20 (7.2%)	20 (6.8%)	40 (7%)
> 20	19 (6.8%)	15 (5.1%)	34 (6%)
Gender			
Male	74 (42%)	42 (22.7%)	116 (30.5%)
Female	102 (58%)	143 (77.3%)	244 (64.5%)
Age (years)			
18 − 24	0	8 (4.5%)	8 (2.2%)
25 − 34	29 (16.4%)	47 (26.3%)	76 (21.3%)
35 − 44	60 (33.9%)	51 (28.5%)	111 (31.2%)
45 − 54	42 (23.7%)	53 (29.6%)	95 (26.7%)
55 − 64	34 (19.2%)	18 (10.1%)	52 (14.6%)
Over 65	12 (6.8%)	2 (1.1%)	14 (3.9%)
Ethnic background			
White	162 (86.6%)	170 (89.9%)	332 (88.3%)
Black/African/Caribbean	0	4 (2.1%)	4 (1.1%)
Mixed/multiple ethnic groups	9 (4.8%)	1 (0.5%)	10 (2.7%)
Asian	9 (4.8%)	12 (6.3%)	21 (5.6%)
Other group	7 (3.7%)	2 (1.1%)	9 (2.4%)
Religion			
No religion	111 (59.7%)	107 (56%)	218 (57.8%)
Christian	58 (31.2%)	66 (34.6%)	124 (32.9%)
Buddhist	1 (0.5%)	5 (2.6%)	6 (1.6%)
Hindu	1 (0.5%)	1 (0.5%)	2 (0.5%)
Jewish	1 (0.5%)	0	1 (0.3%)
Muslim	2 (1.1%)	4 (2.1%)	6 (1.6%)
Sikh	0	5 (2.6%)	5 (1.3%)
Other religion	12 (6.5%)	3 (1.6%)	15 (4%)

Although the sample represented a range of ethnic and religious backgrounds, most participants identified as white (88.3%), and as having no religion (57.8%) (Table 1). Twenty-six per cent of staff indicated having a disability. Of those, 14% said their condition or disability interfered greatly with carrying out their daily activities, 62% said it interfered only a little, and 24% not at all.

### Procedure

A cross-sectional design was adopted in this study supported by quantitative statistical analysis. Data were generated by means of a questionnaire based on convenience sampling. A pilot study was conducted with a small sample of staff to confirm the readability, appropriateness and understanding of the items. Their feedback indicated that few items would benefit from clarification, these items were reformulated to enhance comprehensibility of the questions. The survey initially asked the respondents to confirm if they were staff, their job title and if they worked in a higher education institution in the UK. As the questionnaire was administered online, the questionnaire tool had some unobtrusive methods to detect low quality of responses. The first one was response time, meaning the amount of time spent per page and total amount of time spent completing the questionnaire. Responses that were more than two standard deviations from the media duration of the questionnaire were flagged. The software also had bot detection and protection to flag possible bots. Additionally, participants were prevented from taking the survey more than once, as they were only allowed to make one submission. Patterns of consecutive identical responses provided by the participant (“longstring”) and individual response variability were also flagged. Consequently, participants who responded to items too quickly, had repetitive results (high number of consecutive identical responses) or exhibit low response variability were regarded as invalid. Due to lack of completeness and normative requirements, some responses were excluded. A valid response rate of 87% was obtained.

Participants were recruited through advertisements on organizations’ websites and emails shared by organizations to their staff. Participants were asked to complete an online questionnaire on their experiences and perceptions of their working environment. The completion of the questionnaire took 15 min on average. Participants were then thanked and debriefed. Participation in the study was voluntary and anonymous.

### Measures

#### Organizational fairness and inclusion

Organizational fairness and inclusion were measured via Mor Barak et al’s (1998) instrument. The measure is composed of two dimensions: (a) *Organizational fairness* is assessed by six items regarding perceptions of procedural fairness and discrimination (e.g. “*Managers at my organization give assignments based on the skills and abilities of employees*”). (b) *Organizational inclusion* is composed of four items assessing managerial actions affecting the inclusion or exclusion of members of minority groups (e.g. “*My organization spends enough money and time on diversity awareness and related training “*). Responses were rated on a 6-point scale ranging from *1* = *Strongly disagree* to *6* = *Strongly agree*. All responses were coded so that a high score reflected higher organizational fairness and inclusion. The respondent’s average score on each dimension was computed. Cronbach’s alphas for these dimensions were 0.89 for organizational fairness and 0.64 for organizational inclusion.

#### Psychological safety

Psychological safety was measured by Edmondson’s (1999) 7-item instrument. These items assessed whether the individual felt comfortable to be themselves, bring their ideas forward and make mistakes, or whether there was a threatening environment at work (e.g. “*At my organization, one is free to take risks*”). Participants indicated how accurate the statements were using a 7-point scale ranging from *1* = *Very inaccurate* to *7* = *Very accurate*. Negative items were recoded so that higher scores reflected a safer environment and the average score of the items was computed to create an overall measure of psychological safety. Cronbach's alpha for this measure was 0.85.

#### Work-life balance support

The Hammer’s et al. (2009) *Family Supportive Supervisor behaviors* instrument was used to measure work-life balance support. This 14-item multidimensional scale included four different dimensions of support: emotional support (e.g. “*My manager is willing to listen to my problems in juggling work and non-work life*”), instrumental support (e.g. “*I can depend on my manager to help support me with scheduling conflicts if I need it*”), role-modelling behaviors (e.g. “*My manager is a good role model for work and non-work balance*”), and creative work-family life management (e.g. “*My manager asks for suggestions to make it easier for employees to balance work and non-work demands*”). Items were rated on a scale from *1* = *Strongly disagree to 5* = *Strongly agree*. All items were computed to create an overall score of work-life balance support. Cronbach’s alphas for these measures were 0.90, 0.86, 0.91 and 0.93 for emotional, instrumental support, role-modelling behaviors, and creative work-family life management, respectively.

#### Organizational commitment

Twelve items measuring the organizational commitment as developed by Meyer et al. (1993) were adopted. Two main areas of commitment to the organization were included: affective (e.g. “*I would be very happy to spend the rest of my career with this organization*”) and normative (e.g. “*This organization deserves my loyalty*”). Affective commitment refers to desire-based and normative pertains to obligation-based commitment. Participants’ agreement with each statement was rated on a scale from *1* = *Strongly disagree to 7* = *Strongly agree*. All items were recoded so that higher scores would reflect higher organizational commitment and lower turnover intentions. The respondent’s average score on each dimension was computed. Cronbach’s alphas for these measures were 0.88 and 0.82 for affective and normative commitment, respectively.

#### Sociodemographic characteristics

Participants indicated their gender identity, age, ethnic background, religion, if they had a health problem or disability, a partner, and caring responsibilities.

All measures were selected based on their relevance, constituting well-established scales, that remain valid and adequate in current reality as they continue to be adopted by recent research (e.g. Jiang, 2024; Li et al., 2019; Mogård et al., 2022; Pfajfar et al., 2022; Yu et al., 2022).

## Results

**Fig. 1:**
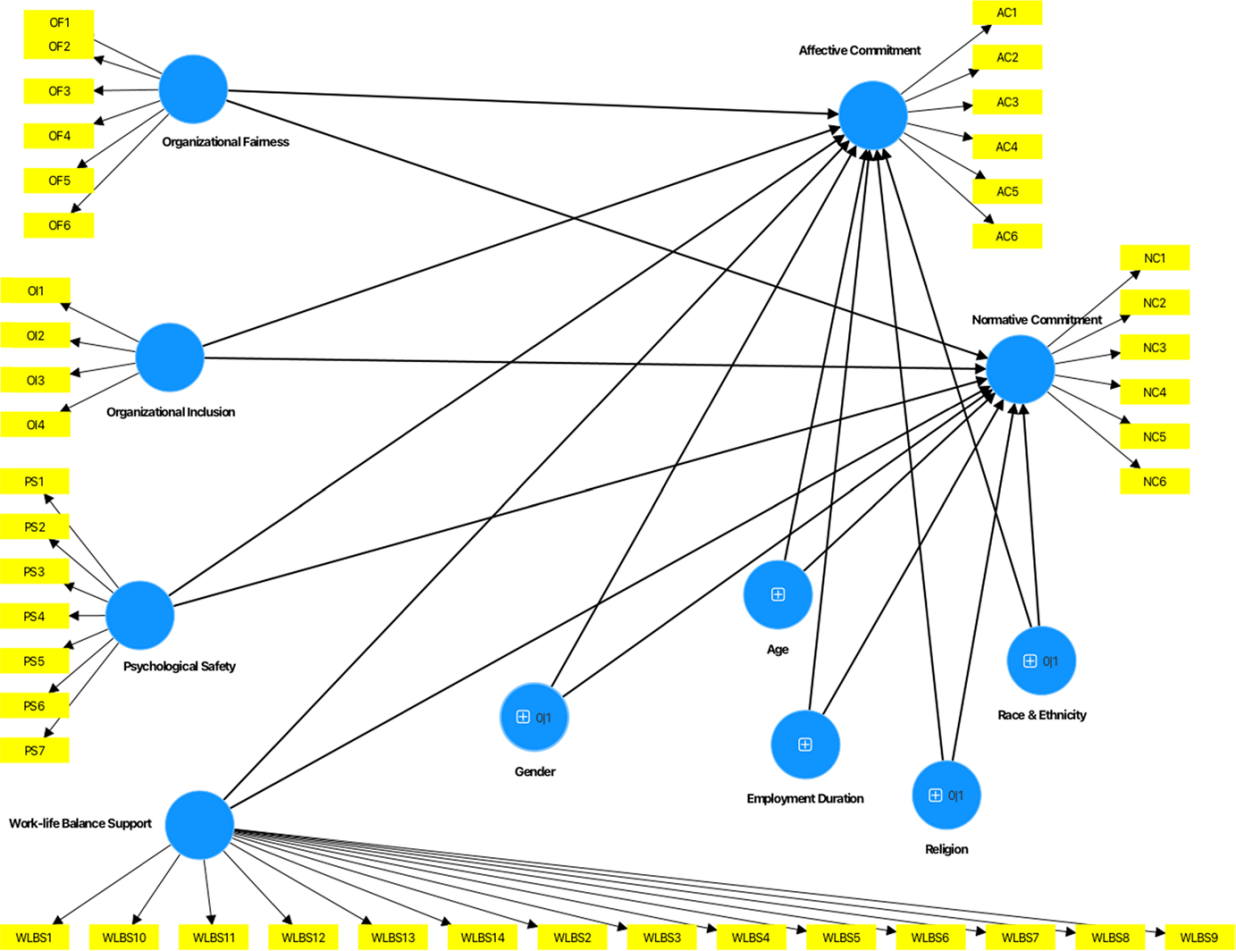
Research model (Complete)

**Fig. 2:**
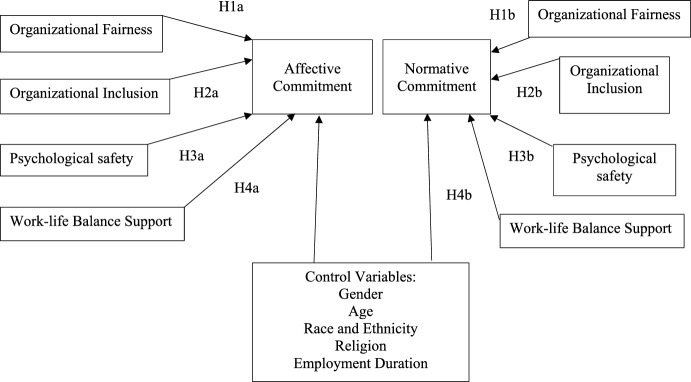
Research model (Hypotheses)

Data was analyzed using the SmartPLS software for Partial Least Squares Structural Equation Modeling (PLS-SEM) to explore the complex interrelationships between the models’ variables (see Fig. 1) and test our hypotheses (Fig. 2). The analysis of the PLS model involved first the evaluation of the measurement model, followed by the evaluation of the structural model. To measure significant differences of the PLS–SEM results for academics and professional services staff, multigroup analysis of the partial least squares (PLS–MGA) was performed. Prior to performing the multigroup analysis, measurement invariance was evaluated using MICOM (Measurement Invariance Assessment) (Henseler et al., 2016). Gender, age, race and ethnicity, employment duration and religion were added to the model as control variables.

### Measurement model

Cronbach alpha and composite reliability values for all constructs were close to or higher than 0.7, indicating construct reliability in all three models (Hair et al., 2019). Average Variance Extracted (AVE) scores were higher than 0.5 (see Table 2), indicating convergent validity (Hair et al., 2019). In all three models, one of the AVE values was not, however composite reliability was higher than 0.6, indicating convergent validity of the construct to be acceptable (Fornell & Larcker, 1981).

**Table 2:** Factor loadings and Cronbach’s alpha reliabilities for study variables

Variables	Items	Factor loadings			Cronbach’s alpha			Composite Reliability			Average Variance Extracted		
		All	ACs	PSS	All	ACs	PSS	All	ACs	PSS	All	ACs	PSS
OF	OF1	0.68	0.67	0.68	0.89	0.89	0.89	0.92	0.92	0.92	0.65	0.65	0.65
	OF2	0.76	0.79	0.73									
	OF3	0.87	0.88	0.87									
	OF4	0.82	0.82	0.83									
	OF5	0.85	0.84	0.86									
	OF6	0.85	0.84	0.86									
OI	OI1	0.74	0.70	0.77	0.64	0.65	0.63	0.79	0.79	0.78	0.48	0.49	0.47
	OI2	0.64	0.70	0.53									
	OI3	0.71	0.70	0.73									
	OI4	0.68	0.68	0.69									
PS	PS1	0.74	0.74	0.73	0.85	0.85	0.86	0.89	0.89	0.89	0.53	0.53	0.54
	PS2	0.81	0.79	0.83									
	PS3	0.73	0.73	0.74									
	PS4	0.69	0.73	0.63									
	PS5	0.70	0.70	0.73									
	PS6	0.60	0.60	0.62									
	PS7	0.81	0.80	0.82									
WLBS	WLBS1	0.84	0.84	0.82	0.97	0.97	0.97	0.97	0.98	0.97	0.73	0.74	0.72
	WLBS2	0.85	0.85	0.83									
	WLBS3	0.81	0.80	0.83									
	WLBS4	0.86	0.87	0.84									
	WLBS5	0.86	0.85	0.86									
	WLBS6	0.81	0.83	0.78									
	WLBS7	0.83	0.82	0.83									
	WLBS8	0.85	0.85	0.85									
	WLBS9	0.89	0.91	0.86									
	WLBS10	0.90	0.91	0.88									
	WLBS11	0.85	0.83	0.87									
	WLBS12	0.85	0.86	0.85									
	WLBS13	0.90	0.90	0.90									
	WLBS14	0.88	0.88	0.89									
AC	AC1	0.79	0.79	0.79	0.88	0.89	0.86	0.91	0.92	0.90	0.63	0.65	0.60
	AC2	0.61	0.66	0.53									
	AC3	0.86	0.87	0.82									
	AC4	0.88	0.88	0.88									
	AC5	0.86	0.85	0.88									
	AC6	0.74	0.78	0.70									
NC	NC1	0.72	0.72	0.72	0.82	0.84	0.80	0.87	0.88	0.85	0.53	0.55	0.49
	NC2	0.63	0.69	0.55									
	NC3	0.67	0.71	0.62									
	NC4	0.82	0.84	0.79									
	NC5	0.73	0.73	0.76									
	NC6	0.76	0.77	0.75									

*ACs* Academics, *PSS* professional services staff, *OF* organizational fairness, *OI* organizational inclusion, *PS* psychological safety, *WLBS* work-life balance support, *RE* religion, *AC* affective commitment, *NC* normative commitment

Finally, discriminant validity was accessed by the Heterotrait-monotrait ratio of correlations (HTMT) and Fornell and Larcker’s (1981). As it can be seen in Table 3 the square root of AVEs and correlations of all constructs are more than the diagonal correlation values for all models, and in Table 4 the majority of HTMT values were below than 0.85, indicating good discriminant validity. The models were also assessed for collinearity using the index variance inflation factor (VIF). All VIF values were within the threshold value of 5.0, indicating the absence of multicollinearity in the model and no contamination of Common Method Bias (O’Brien, 2007).

**Table 3:** Fornell-Larcker Criterion

	All						Academics						Professional Services Staff					
Variables	1	2	3	4	5	6	1	2	3	4	5	6	1	2	3	4	5	6
1. Organizational fairness	0.81						0.81						0.81					
2. Organizational inclusion	0.65	0.69					0.62	0.70					0.70	0.69				
3. Psychological safety	0.69	0.64	0.73				0.70	0.62	0.73				0.67	0.67	0.74			
4. Work-life balance support	0.53	0.47	0.59	0.86			0.56	0.46	0.63	0.86			0.48	0.49	0.54	0.85		
5. Affective commitment	0.46	0.42	0.53	0.47	0.80		0.47	0.43	0.57	0.46	0.81		0.45	0.42	0.47	0.47	0.78	
6. Normative commitment	0.42	0.37	0.43	0.45	0.67	0.72	0.50	0.39	0.52	0.46	0.70	0.74	0.31	0.33	0.31	0.46	0.62	0.70

**Table 4:** Heterotrait-monotrait ratio of correlations

	All						Academics						Professional services staff					
Variables	1	2	3	4	5	6	1	2	3	4	5	6	1	2	3	4	5	6
1. Organizational fairness	–						–						–					
2. Organizational inclusion	0.85	–					0.81	–					0.90	–				
3. Psychological safety	0.78	0.87	–				0.80	0.84	–				0.76	0.89	–			
4. Work-life balance support	0.56	0.58	0.65	–			0.60	0.58	0.70	–			0.51	0.58	0.58	–		
5. Affective commitment	0.49	0.54	0.58	0.49	–		0.49	0.54	0.61	0.48	–		0.50	0.54	0.51	0.50	–	
6. Normative commitment	0.47	0.47	0.48	0.48	0.76	–	0.56	0.50	0.58	0.48	0.77	–	0.34	0.41	0.34	0.48	0.74	**–**

### Preliminary analysis

Means, Standard Deviations, and Pearson correlations among organizational fairness, inclusion, psychological safety, work-life balance support, affective and normative commitment are presented in Table 5. Correlation analyses were conducted on the full sample separately for academic and professional services staff.

**Table 5:** Relationships (mean (M), standard deviation (SD) and Pearson correlations) between study measures for both academic and professional services staff

							Professional services staff	
Variables	1	2	3	4	5	6	*M*	*SD*
1. Organizational fairness	*–*	0.68***	0.68***	0.51***	0.49***	0.30***	4.31	1.23
2. Organizational inclusion	0.66***	–	0.68***	0.46***	0.43***	0.30***	3.51	1.04
3. Psychological safety	0.70***	0.65***	–	0.54***	0.48***	0.29***	4.38	1.21
4. Work-life balance support	0.56***	0.44***	0.59***	–	0.48***	0.44***	3.61	1.12
5. Affective commitment	0.45***	0.44***	0.56***	0.42***	–	0.61***	4.39	1.39
6. Normative commitment	0.50***	0.46***	0.56***	0.47***	0.69***	–	3.71	1.30
Academic *M*	3.92	3.28	4.01	3.25	3.93	3.42		
Academic* SD*	1.27	1.00	1.22	1.14	1.61	1.47		

Higher scores on all measures reflect higher levels of the construct. Correlations for professional services staff are presented above the diagonal; for academics, below the diagonal

***p < 0.001

Organizational fairness was moderately correlated with organizational inclusion for both academic (*r* = 0.66, *p* < 0.001) and professional services staff (*r* = 0.68, *p* < 0.001) (Table 5). Regarding the relationship between organizational fairness and psychological safety, the findings showed that correlations were moderate to strong:* r* = 0.70, *p* < 0.001 for academic staff and *r* = 0.68, *p* < 0.001 for professional service staff (Table 5). Similarly, the correlations between organizational inclusion and psychological safety were moderate: *r* = 0.65, *p* < 0.001 for academic staff and *r* = 0.68, *p* < 0.001 for professional service staff (Table 5).

Results in Table 2 demonstrate that staff’s perceptions of managerial support for work-life balance were positively correlated with organizational fairness (*r* = 0.56, *p* < 0.001 for academic staff and *r* = 0.51, *p* < 0.001, for professional service staff) and inclusion (*r* = 0.44, *p* < 0.001 for academic staff and *r* = 0.46, *p* < 0.001 for professional service staff).

Regarding the relationship between psychological safety and work-life balance support, the findings indicate that correlations were moderate, for both academic and professional services staff (*r* = 0.59, *p* < 0.001 and *r* = 0.54, *p* < 0.001, respectively).

The correlations between affective commitment, organizational fairness and inclusion, psychological safety and support for work-life balance were low to moderate for both academic and professional service staff, with correlation coefficients ranging from 0.42 to 0.56.

Finally, Table 5 shows that normative commitment, organizational fairness and inclusion, psychological safety and support for work-life balance were low to moderately correlated, for both academic and professional service staff, with correlation coefficients ranging from 0.29 to 0.69.

**Fig. 3:**
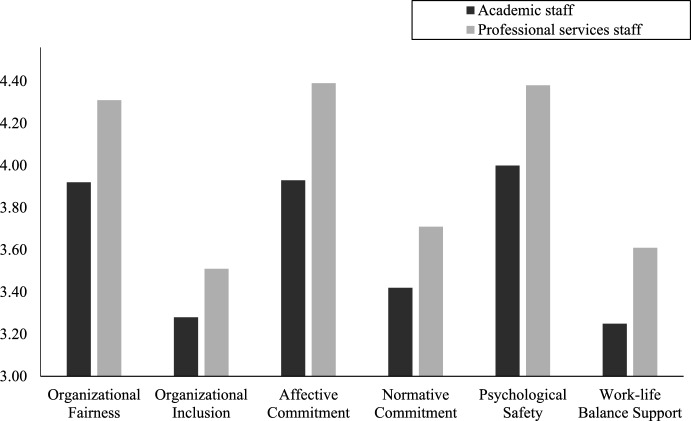
Comparison of mean scores for each study measure between academic and professional services staff

To explore the differences between academic and professional services staff’s perceptions of their organizational environment, independent sample *t*-tests (Fig. 3) were conducted.

Comparisons between professional service and academic staff’s perceptions of organizational fairness revealed that the latter (*M* = 3.92, *SD* = 1.27) perceived organizational processes to be less fair and non-discriminatory than professional services staff (*M* = 4.31, *SD* = 1.23)*,* (*t* (384) = − 3.05, *p* = 0.002). Academic staff (*M* = 3.28, *SD* = 1.00) also perceived managerial actions to be less inclusive of minority groups than professional services staff (*M* = 3.51, *SD* = 1.04), (*t* (382) = − 2.19, *p* = 0.029).

Comparison in organizational commitment between academic and professional services staff suggested that academic staff (*M* = 3.93, *SD* = 1.61) indicated lower levels of affective commitment than professional services staff (*M* = 4.39, *SD* = 1.39), (*t* (353) = − 2.92, *p* = 0.004), and also lower normative commitment (*M* = 3.42, *SD* = 1.47; *M* = 3.71, *SD* = 1.30, academic and professional services staff, respectively), (*t* (357) = − 1.98, *p* = 0.048).

When comparing psychological safety levels among academic and professional services staff, it was observed that academic staff (*M* = 4.00, *SD* = 1.22) scored significantly lower on psychological safety than professional services staff (*M* = 4.38, *SD* = 1.21), (*t* (391) = − 3.07, *p* = 0.002) (Fig. 3). This means that academic staff felt less safe to take interpersonal risks, less comfortable being themselves and making mistakes than professional services staff.

Analysis of how supported university staff feel in terms of their work-life balance, revealed that academic staff (*M* = 3.25, *SD* = 1.14) overall felt less supported in their work-life balance than professional services staff (*M* = 3.61, *SD* = 1.12), (*t* (380) = − 3.09, *p* = 0.002). Academic staff (*M* = 3.44, *SD* = 1.17) also felt less supported emotionally by their managers than professional services staff (*M* = 3.80, *SD* = 1.13), (*t* (382) = − 3.05, *p* = 0.002); and instrumentally, (*M* = 3.23, *SD* = 1.18; *M* = 3.63, *SD* = 1.14, for academic and professional services staff, respectively), (*t* (382) = − 3.32, *p* = 0.001).

Regarding managerial behavior, academic staff (*M* = 3.15, *SD* = 1.26) considered that managers exhibited less role model behaviors when balancing work and life issues than professional services staff (*M* = 3.43, *SD* = 1.20), (*t* (382) = − 2.23, *p* = 0.026). Similarly, academic staff (*M* = 3.17, *SD* = 1.18) also viewed their managers’ work-life balance solutions as less creative than professional services staff (*M* = 3.51, *SD* = 1.21), (*t* (383) = − 2.80, *p* = 0.005).

### Structural model

**Fig. 4:**
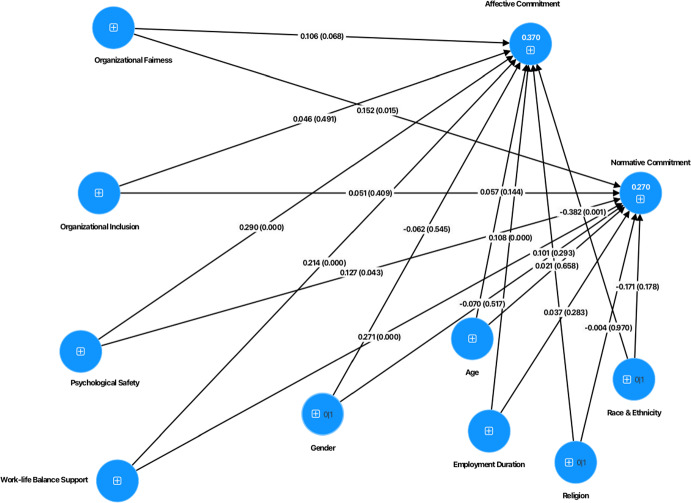
Structural model (Complete)

To test the proposed hypotheses, the suggested model was evaluated through PLS-SEM, the values of the path coefficients (*β*) and the explained variance (*R*^*2*^) were considered for the full dataset with 5000 iterations of resampling (see Table 6, Fig. 4 for complete model). The results of the structural model on the full dataset show that the path coefficients were significant and positive, organizational fairness positively influences staff’s normative commitment (*β* = 0.152, *p* = 0.015), providing support for hypothesis H1b.

**Table 6:** Structural model evaluation

	Path coefficient			*t-value*			CIs			f^2^ effect size		
	All	ACs	PSS	All	ACs	PSS	All	ACs	PSS	All	ACs	PSS
OF → AC	0.106	0.056	0.176	1.828	0.718	1.944	[− 0.01; 0.22]	[− 0.09; 0.21]	[0.01; 0.37]	0.008	0.002	0.020
OF → NC	0.152*	0.212**	0.076	2.438	2.538	0.771	[0.02; 0.27]	[0.05; 0.38]	[-0.12; 0.27]	0.013	0.029	0.003
OI → AC	0.046	0.049	0.042	0.689	0.554	0.479	[− 0.08; 0.18]	[− 0.12; 0.22]	[− 0.13; 0.22]	0.002	0.002	0.001
OI → NC	0.051	0.027	0.104	0.825	0.339	0.993	[− 0.07; 0.18]	[− 0.13; 0.18]	[− 0.11; 0.31]	0.002	0.001	0.006
PS → AC	0.290***	0.399***	0.171	4.984	5.114	1.840	[0.18; 0.41]	[0.24; 0.55]	[− 0.01; 0.36]	0.054	0.102	0.019
PS → NC	0.127*	0.236**	-0.010	2.021	2.887	0.097	[0.01; 0.25]	[0.08; 0.40]	[− 0.20; 0.19]	0.009	0.032	0.000
WLBS → AC	0.214***	0.142	0.271***	3.945	1.930	3.253	[0.11; 0.32]	[− 0.01; 0.29]	[0.10; 0.42]	0.045	0.019	0.073
WLBS → NC	0.271***	0.160*	0.370***	5.287	2.198	4.994	[0.17; 0.37]	[0.02; 0.30]	[0.23; 0.52]	0.062	0.022	0.117

*ACs* Academics, *PSS* professional services staff, *OF* organizational fairness, *OI* organizational inclusion, *PS* psychological safety, *WLBS* work-life balance support, *RE* religion, *AC* affective commitment, *NC* normative commitment

*p < 0.05 , **p < 0.01 , ***p < 0.001

On the other hand, the relationships between organizational inclusion and affective (*Β* = 0.05, *p* = 0.491) and normative commitment (*β* = 0.051, *p* = 0.410) showed path coefficients that were positive but not significant, meaning that results did not support H2a and H2b.

Additionally, the path coefficients showed that psychological safety positively influences affective (*β* = 0.290, *p* < 0.001) and normative commitment (*β* = 0.127, *p* = 0.043), and work-life balance support positively influences affective (*β* = 0.214, *p* < 0.001) and normative commitment (*β* = 0.271, *p* < 0.001), providing support for hypotheses H3a, H3b, H4a and H4b, respectively. Furthermore, the path coefficients showed that from the control variables only employment duration, race and ethnicity had a positive influence on affective commitment (*β* = 0.108, *p* < 0.001 and *β* = -0.382, *p* = 0.001, respectively). This meant that the longer staff worked at the university the more they developed an emotional attachment to the organization. Additionally, white members of staff expressed stronger levels of affective commitment compared to staff from non-white/ethnic minority backgrounds.

To assess the structural model, its explanatory capacity is evaluated using the *R*^2^ value, which reflects the explained variance of the dependent constructs (Hair et al., 2019). The *R*^2^ values obtained for the complete model were 0.37 for affective commitment (r^2^ adjusted = 0.361) and 0.27 for normative commitment (r^2^ adjusted = 0.260), indicating a moderate predictive capacity of the model presented.

**Fig. 5:**
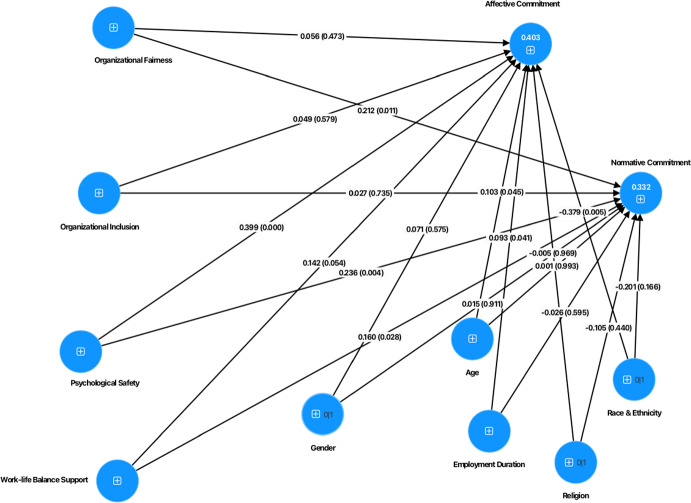
Structural model (Academics)

**Fig. 6:**
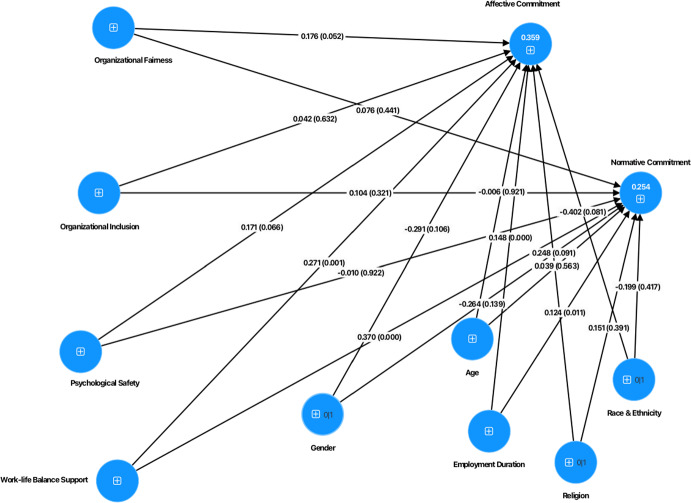
Structural model (Professional Services Staff)

The results for each specific role, revealed that for academics, organizational fairness positively influences normative commitment (*β* = 0.212, *p* = 0.011), while psychological safety positively influences affective (*β* = 0.399, *p* < 0.001) and normative commitment (*β* = 0.236, *p* = 0.004). Consistent with the results of the total sample, work-life balance support has a positive impact on affective (*β* = 0.271, *p* = 0.001) for professional services staff and normative commitment (*β* = 0.160*, p* = 0.028; *β* = 0.370, *p* < 0.001) for academics and professional services staff respectively (see Fig. 5 for the model for academics and Fig. 6 model for professional services staff). The values of *R*^2^ obtained were 0.403 (r^2^ adjusted = 0.388), 0.359 (r^2^ adjusted = 0.339) for affective commitment and 0.332 (r^2^ adjusted = 0.315), 0.254 (r^2^ adjusted = 0.231) for normative commitment for academics and professional services staff respectively, indicating a moderated predictive capacity of the two models.

Effect sizes (*f*^2^) shown in Table 6 were mostly small. Effect of work-life balance support on affective and normative commitment revealed a greater effect for professional services staff compared with academics. The out-of-sample predictive power of the models revealed positive Q^2^ predictive values for affective (0.330, 0329, 0.286 for complete, academic and professional services staff models) and normative commitment (0.228, 0.253, 0.165 for complete, academic and professional services staff models), indicating moderate out-of-sample predictive power assessment. The models were checked for goodness of fit criteria using standardized root-mean-square residual (SRMR) obtaining the values of 0.054, 0.061 and 0.063, for complete model, academics and professional services staff respectively and within the cut-off value of 0.08 (Henseler et al., 2015).

### Multigroup analysis (MGA)

To further explore role differences, a multigroup analysis was performed using the Measurement Invariance Assessment (MICOM) procedure (see Table 7). MICOM ensures that the differences among groups of respondents are not due to the different meanings of the latent variables. This process facilitates the comparison of group parameters and allows to determine the presence or absence of partial, full or no measurement invariance (Henseler et al., 2016). It involved three steps: (1) configural invariance, (2) compositional invariance, and (3) the equality of composite mean values and variances (Henseler et al., 2016). First, configural invariance was established. The results of the compositional invariance evaluation for Step 2 were partly established for organizational fairness, psychological safety, religion, affective commitment, normative commitment.

**Table 7:** Results of invariance measurement testing using permutation

	Step 1 configural invariance	Compositional invariance			Equal mean values			Equal variances			Measurement invariance
		C = 1	CIs	Equal	Diff	CIs		Diff	CIs		
Organizational fairness	Yes	0.999	[0.999, 0.997]	Yes	0.007	[− 0.150, 0.157]	No	− 0.006	[− 0.254, 0.249]	Yes	Partial
Organizational inclusion	Yes	0.993	[0.990, 0.973]	Yes	0.005	[− 0.149, 0.163]	Yes	− 0.002	[− 0.259, 0.252]	Yes	Full
Psychological safety	Yes	0.997	[0.998, 0.995]	Yes	0.004	[− 0.153, 0.162]	No	− 0.002	[− 0.252, 0.267]	Yes	Partial
Work-life balance support	Yes	0.999	[1.000, 0.999]	No		(not evaluated)					
Age	Yes	1.000	[1.000, 1.000]	No		(not evaluated)					
Gender	Yes	1.000	[1.000, 1.000]	No		(not evaluated)					
Employment duration	Yes	1.000	[1.000, 1.000]	No		(not evaluated)					
Race and ethnicity	Yes	1.000	[1.000, 1.000]	No		(not evaluated)					
Religion	Yes	1.000	[1.000, 1.000]	Yes	0.000	[− 0.60, 0.057]	No	− 0.002	[− 0.124, 0.118]	Yes	Partial
Affective commitment	Yes	0.999	[0.999, 0.996]	Yes	0.002	[− 0.146, 0.160]	No	0.004	[− 0.231, 0.249]	No	Partial
Normative commitment	Yes	0.999	[0.996, 0.991]	Yes	0.004	[− 0.153, 0.160]	Yes	− 0.001	[− 0.234, 0.235]	No	Partial

Except for organizational inclusion (where full invariance was established), results of step 3 showed that partial invariance of the measurement has been established for all the other measures.

After completing the MICOM procedure, Multigroup Analysis was carried out (Table 8), to assess the similarities and differences of path coefficients across the two groups. For academics path coefficients were significant for the relations between organizational fairness and normative commitment, psychological safety, affective and normative commitment. This indicated that academics’ normative commitment was more influenced by organizational fairness than in the case of professional services staff. Furthermore, affective and normative commitment are more strongly affected by psychological safety among academics than professional support staff.

**Table 8:** Multigroup analysis

	Path coefficient		*t-value*		*p-value*	
	ACs	PSS	ACs	PSS	ACs	PSS
OF → AC	0.056	0.176	0.711	1.954	0.473	0.052
OF → NC	0.212	0.076	2.550	0.783	0.011*	0.441
OI → AC	0.049	0.042	0.545	0.483	0.579	0.632
OI → NC	0.027	0.104	0.335	0.988	0.735	0.321
PS → AC	0.399	0.171	5.112	1.828	0.000***	0.066
PS → NC	0.236	-0.010	2.820	0.099	0.004**	0.922
RE → AC	-0.005	0.248	0.037	1.695	0.969	0.091
RE → NC	-0.105	0.151	0.754	0.867	0.440	0.391

*ACs* Academics, *PSS* professional services staff, *OF* organizational fairness, *OI* organizational inclusion, *PS* psychological safety, *WLBS* work-life balance support, *RE* religion, *AC* affective commitment, *NC* normative commitment

*p < 0.05, **p < 0.01, ***p < 0.001

## Discussion

This study aimed to explore how organizational fairness and inclusion, psychological safety and support for work-life balance influence organizational commitment and vary among academic and professional services staff. Our theoretical framework and empirical strategy offer a distinct approach to prior literature by focusing on how organization related dimensions influence commitment analyzing academics and professional services staff. The results lead to a better understanding of organizational practices and culture and its impact on university staff’s affective and normative commitment.

The findings revealed that overall academics manifested lower levels of affective and normative commitment, psychological safety and felt less supported in their work-life balance than their professional services colleagues, adding to previous literature (e.g. Fontinha et al., 2019; Johnson et al., 2019; Tytherleigh et al., 2005) and giving insight into the differential impact organizational culture has on academics and professional staff, advancing our understanding in the comparison of experiences among both roles. This is perhaps indicative of the higher self-centricity observed (and necessary) in the academic role. The drive to develop themselves as world-leaders in their field, developing a professional identity that is independent of the organization. Furthermore, with a growing number of academic staff being employed on fixed-term contracts, and academic careers becoming less stable (Catano et al., 2010; HESA, 2021), moving from organization to organization is becoming a defining characteristic of an academic career, and will lead to fewer opportunities, and less commitment, to remain at a single institution for any length of time. As service providers, on the other hand, professional services staff are more organizational-centric, and perhaps see themselves as working towards improving the organization (e.g. its policies and practices), and developing themselves within, and dependent upon, the organization.

In line with our first set of hypotheses, the findings showed that organizational fairness positively influences staff’s normative commitment. However, the effects of organizational fairness on normative commitment tended to be higher among academics than professional services staff. This suggests that the perception of procedural fairness and discrimination, despite being an important element for both groups of staff, impacts academics loyalty and felt obligation to remain and reciprocate organizational investments to a higher extent. Our research confirms the importance of fairness as crucial to resource preservation, enhancement, and leading to higher commitment (e.g. Au & Ahmed, 2016; Clark et al., 2017; Janssen et al., 2010; Rofcanin et al., 2017). It further makes an important new contribution to the literature by illustrating that perceiving processes and outcomes as impartial has higher impact on how academics (compared to professional services staff) feel gratitude towards, willingness and obligation to continue working at the university and how management should invest in having impartial, fair and transparent procedures and practices to retain academic staff specifically.

Interestingly and unexpectedly, organizational inclusion did not significantly influence staff’s affective or normative commitment. In line with previous research, our study found that organizational inclusion was associated with higher levels of organizational commitment (e.g. Findler et al., 2007; Mor Barak et al., 2016), however organizational inclusion did not influence the two distinct types of commitment explored. Recent research (Chung et al., 2020; Innstrand & Grødal, 2021) suggests that overall justice is not significantly related to inclusion, demonstrating that the two concepts are distinct and might influence organizational commitment in different ways. This finding might also reflect the lack of diversity in the sample, that might not personally experience the barriers associated with being part of a minoritized group and therefore, organizational inclusion might not be as prominent to elicit commitment.

The findings also supported our third set of hypotheses, indicating that psychological safety positively influence staff’s affective and normative commitment. Our results add to previous literature by confirming the relationship between psychological safety and organizational commitment (e.g. Brimhall et al., 2017; Edmonson, 2018; Frazier et al., 2017) and add original contributions by differentiating such relationship in distinct forms of commitment (affective and normative). Furthermore, affective and normative commitment were more strongly affected by psychological safety among academics than professional services staff. This is a novel aspect that should be highlighted. An argument could be made that psychological safety is a crucial element of the organizational environment and vital to influence academics’ emotional connection, identification, as well as loyalty and felt obligation to remain with and contribute to the organization. Psychological safety might be perceived by academics as the university caring and valuing them which in turn impacts building stronger emotional bond with the organization, making it an essential element to retain academic staff in particular.

Finally, work-life balance support positively influenced staff’s affective and normative commitment, confirming our fourth set of hypotheses. This finding adds to previous research that demonstrated the impact of work-life balance support on academic staff’s commitment (Fontinha et al., 2018) and brings novel inputs by showing the impact of work-life balance support on academic and professional services staff and on both types of commitment (affective and normative). Work-life balance support appears to be an equally important dimension to drive affective and normative commitment from both academics and professional services staff.

### Theoretical implications

Drawing on conservation of resources theory, the current study offers a deeper understanding of the links between organizational factors and staff’s commitment and highlights the importance of including such factors when exploring organizational affective and normative commitment. While most of the studies to date have focused on either analyzing the experiences of academic staff or professional services staff separately, or have made limited comparisons between the two, the current study analyses the operation and differential impact of organizational practices and priorities comparing academic and professional services staff in a single comprehensive design. Therefore, findings provide new insight into how different dimensions of the organizational environment influence different types of commitment among academics and professional services staff. Results indicate that organizational fairness, psychological safety and support for work-life balance can be seen by staff as the organization valuing and caring for their well-being which in turn activates a need to reciprocate and enhance commitment. Therefore, the proposed model explains the processes within it, in the specific context of higher education in the UK.

While there is ample research on organizational commitment at a macrolevel, few studies have included social psychological mechanisms (e.g. organizational fairness, inclusion, psychological safety) and explored their role on higher education staff’s affective and normative commitment. The proposed distinction of both types of commitment is supported by the results and echoes earlier studies that highly the importance of this differentiation as normative and affective commitment are influenced by different factors (Al-bdour et al., 2010; Brammer et al., 2007; Meyer et al., 2002; Mory et al., 2017).

### Practical implications

As higher education institutions face the growing challenge of employing and retaining talent, this study offers useful information to Human Resources professionals, university administration and governance as our results open new pathways for organizations to promote affective and normative commitment. First, improvement of work-life balance support can further enhance commitment from academics and professional services staff. Together with organizational culture, managers are instrumental in providing the support needed for work-life balance (Dikkers et al., 2004; Thompson & Prottas, 2006). They need to be able to tailor their approaches to the context and staff for whom they are responsible and develop the strategies and practices that best benefit their teams. Equally, they need to be aware of their own attitudes and practices, and the importance of exhibiting good role model behaviors towards work-life balance. Organizations are encouraged to adopt programs that focus on supporting work-life balance and provide training for managers and leaders at all levels so they can develop skills to enhance support for work-life balance and consequently enhance affective and normative commitment among staff.

Second, managers and leaders at all levels of the organization cannot overlook the influence of organizational fairness and psychological safety on commitment. It is essential that management’s policies and procedures are transparent and equitable, and managers do not give preferential treatment in hiring or promoting staff, especially if organizations want to retain academics through evoking loyalty and a sense of obligation to remain with and contribute to the organization. Moreover, organizations should not underestimate the role of leaders in shaping psychologically safe environments and should invest in providing training and coaching to managers, developing inclusive leadership competencies, and ensuring they develop skills on how to foster and sustain psychologically safe environments. This is particularly relevant for educing an emotional attachment and a sense of obligation to remain in the organization among academics. Therefore, organizations should focus their efforts on growing psychological safety when wanting to increase commitment and retention among their academic staff.

Additionally, practitioners should include a measure of organizational fairness, psychological safety and work-life balance support in their staff surveys, to enable the comparison of responses over time, measure progress and evaluate the impact of their Equality, Diversity and Inclusion initiatives.

Finally, the current study has implications for the governance of the universities under analysis and organizations in general. Effective governance requires engaging actors to be involved in developing a shared vision and culture of the organization. As common values and cultural governance are crucial factors for deeper cultural change (Niedlich et al., 2020; Viegas et al., 2016), it is essential that all staff is involved in developing and implementing policy actions in defense of fairness, psychological safety and support for work-life balance, and organizations do not just carry out short-term activities that do not lead to real cultural change. Enhanced affective and normative commitment influence staff’s identification with the organization, job satisfaction and potentially impact performance, benefiting the organization they work for and increase retainment. increasing psychological safety and support for work-life balance to increase organizational affective and normative commitment.

## Conclusion

Overall, the results of this study bring important contributions by generating further evidence of the experience and perceptions of different groups of people in organizations, enhancing our understanding of how practices can promote individual integration and contribute to systemic change in the higher education context. It begins to unravel the mechanisms through which organizational fairness, inclusion, psychological safety and work-life balance support influence commitment to the organization. By examining role differences in organizational environment, we gain a better understanding of the processes that influence organizational commitment and staff well-being, and the differential impact across academic and professional services roles.

Due to the cross-sectional design of the study, no definitive causal conclusions can be made concerning the relationships between organizational environment and commitment. Therefore, future research would benefit from collecting data on the overall employment duration and using a longitudinal design, allowing for a better understanding of how the organizational environment influences, and is influenced by, commitment. Future studies would benefit from the use of in-depth interviews with staff, to explore in detail their perceptions of the organizational environment, challenges and factors that might contribute to their commitment to the organization.
